# Engaging plasticity: Differentiation therapy in solid tumors

**DOI:** 10.3389/fphar.2022.944773

**Published:** 2022-08-11

**Authors:** Neta Bar-Hai, Dana Ishay-Ronen

**Affiliations:** ^1^ Cancer Research Center, Oncology Institute, Chaim Sheba Medical Center, Tel-Hashomer, Israel; ^2^ Affiliated with Sackler Faculty of Medicine, Tel Aviv University, Tel Aviv, Israel

**Keywords:** cancer cell plasticity, EMT, differentiation therapy, trans-differentiation, TGFb signaling, solid tumors

## Abstract

Cancer is a systemic heterogeneous disease that can undergo several rounds of latency and activation. Tumor progression evolves by increasing diversity, adaptation to signals from the microenvironment and escape mechanisms from therapy. These dynamic processes indicate necessity for cell plasticity. Epithelial-mesenchymal transition (EMT) plays a major role in facilitating cell plasticity in solid tumors by inducing dedifferentiation and cell type transitions. These two practices, plasticity and dedifferentiation enhance tumor heterogeneity creating a key challenge in cancer treatment. In this review we will explore cancer cell plasticity and elaborate treatment modalities that aspire to overcome such dynamic processes in solid tumors. We will further discuss the therapeutic potential of utilizing enhanced cell plasticity for differentiation therapy.

## 1 Introduction

Plasticity in biology is viewed as the capacity to adapt and survive under changes. Plasticity in a cell serves as an escape mechanism enabling the cell to adapt to fluctuating conditions. Escape requires the involvement of many cellular components: cytoskeleton rearrangements, transcriptional and post-transcriptional changes, and even of altered cellular function. These global changes take place under differentiation, being one domain in which plasticity is demonstrated. A stem cell can always differentiate, which means that it always has a powerful escape mechanism at hand. Another important example of cell plasticity in development and pathologic responses is the process of EMT. During EMT epithelial cells undergo a dedifferentiation process and progressively lose epithelial phenotype and function (Tiwari et al., 2012; Nieto, 2013; Lamouille et al., 2014). In cancer, EMT and cancer cell plasticity contribute to malignant progression and the development of drug resistance (Berx et al., 2007; Puisieux et al., 2014; Brabletz et al., 2018; Boumahdi and de Sauvage, 2020). An additional substantial characteristic of cancer cells is their differentiation potential which was introduced by G.B. Pierce (PIERCE and DIXON, 1959; Arechaga, 2003) and fueled the quest for cancer differentiation treatment. The steering of cancer cells into a benign direction was successfully achieved in the revolution of acute promyelocytic leukemia (APL) treatment (Wang and Chen, 2008; Coombs et al., 2015). In the case of solid tumors, the application of differentiation-based therapy is rather obscure (Vogelstein et al., 2013; de Thé, 2018). The different approaches to overcome cancer plasticity and the complexity confronting tumor survival dynamics will be described in this review. The perception of differentiation therapy as reversing of cell plasticity will be discussed as well.

## 2 Cancer plasticity

**FIGURE 1:**
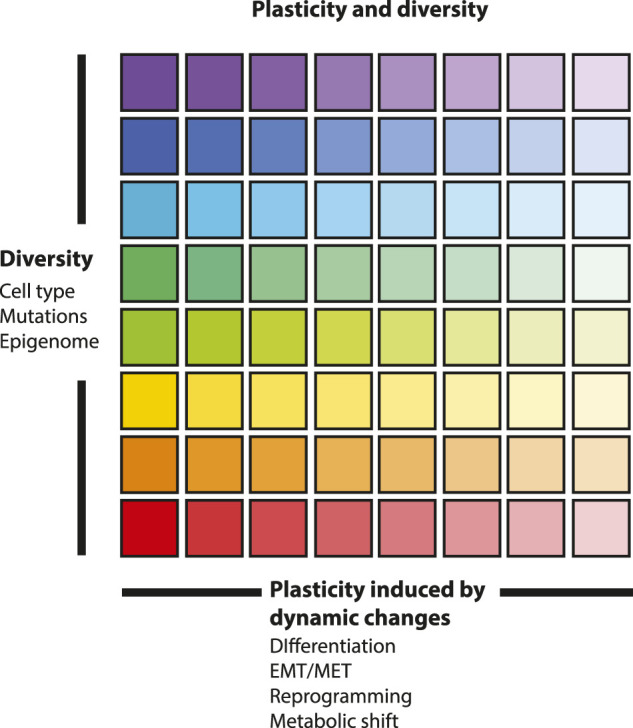
Plasticity and diversity in a cell population. This figure symbolizes the inherent plasticity of every differentiated state and of every cell type on the vertical axis. Plasticity, induced by dynamic changes, is represented on the horizontal axis. Differentiated states are on the left where different colors are marked, and dedifferentiated and stem cell-like states are on the right where colors are light. Cell diversity includes cell identity, mutations and epigenetic landscape of cells creating cellular heterogeneity. Plasticity is triggered by external signals from the tumor microenvironment and is exhibited in the varying response of cells depending on cell state and location, thus creating a variety of phenotypic changes.

Analogously to animal and plant life, the survival of a cell population, is achieved through its inherent variations (epigenetic modifications, mutations, epistasis), and its capacity to undergo adaptation induced by dynamic changes. In cancer, specific mutations can induce cell type transitions and enhance cell plasticity contributing to cancer heterogeneity (Koren et al., 2015; Van Keymeulen et al., 2015). Cancer cell plasticity is exhibited in the varying responses of cells depending on cell state and location, thus creating a variety of phenotypic changes (Figure 1). In this section we focus on cancer cell plasticity and its impact on cancer progression and drug resistance.

### 2.1 Epithelial-mesenchymal transition in cancer

**FIGURE 2:**
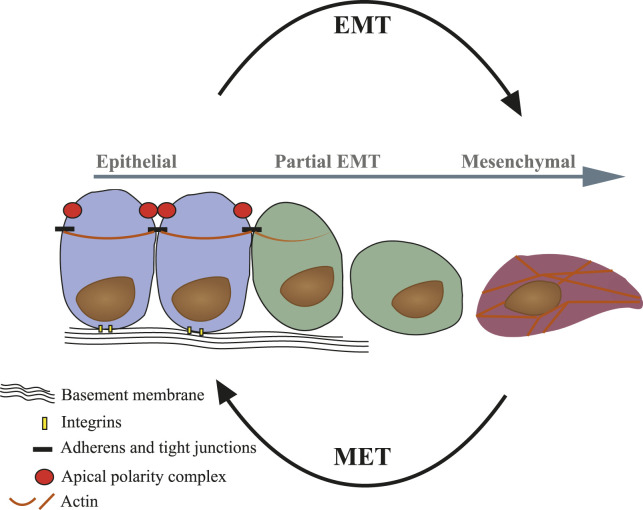
Representation of structural and cellular changes during EMT/MET. Epithelial cells (blue) exhibit apical-basal polarity and cortical actin organization. Epithelium comprises tightly packed and functionally synchronized epithelial cells connected to each other via cell-cell junctions and are anchored to the basement membrane *via* integrins. Upon EMT, cells lose epithelial characteristics and become dedifferentiated single cells (partial EMT—green). The full conversion gives rise to mesenchymal-like cells (purple) with front-rear polarity and actin stress fibers formation.

Epithelial-mesenchymal transition (EMT) is a biological program during which differentiated epithelial cells lose their epithelial characteristics such as cell-cell adhesions and apical-basal polarity and gain migratory properties (Box 1—Hallmarks of EMT). EMT is considered a process of dedifferentiation, rather than a process of trans-differentiation of epithelial into a mesenchymal cell or a fibroblast (Berx et al., 2007). The full process of EMT is complex and prolonged in time. At any point during the process, if the signal is removed, the cells will revert to the epithelial state through a mesenchymal-to-epithelial-transition (MET) (Lamouille et al., 2014). However, the plasticity acquired during this process seems to be reduced if the signal remains consistent, bringing about a stabilization of mesenchymal state (Zhang et al., 2014). When stabilized in the mesenchymal state it is hard to distinguish between EMT-derived cells and a fibroblast. Yet, a fibroblast is less likely to undergo MET or any other cell type transition. Partial EMT refers to any state observed between EMT induction and full mesenchymal state, and can include markers of both epithelial and mesenchymal cells at varying levels (Nieto, 2013). These cells are unstable and will quickly revert to the epithelial state once the external stimulus is removed (Figure 2). EMT and MET are of central importance during embryogenesis and organ development, facilitating cell migration and cell type transitions to allow crucial for normal development (Nieto, 2013). Cancer cells may adapt by hijacking developmental programs such as EMT to enhance cell plasticity (Puisieux et al., 2014; Brabletz et al., 2018). Thus, EMT contributes to cancer heterogeneity, dissemination and the development of drug resistance. In fact, EMT facilitates cancer cells’ escape both literally, with the transfer from stationary to motile cells, and also figuratively, with the acquisition of dynamic response capabilities. In recent years, our perception of EMT in cancer progression has evolved from a simple binary model into a multi-step model with intermediate transition states on the spectrum between epithelial to mesenchymal, characterized by high plasticity (Polyak and Weinberg, 2009; Tsai and Yang, 2013; Nieto et al., 2016; Aiello et al., 2018; Pastushenko et al., 2018; Yang et al., 2020). The *in vivo* identification of partial EMT in cancer has been technically challenging (Trimboli et al., 2008; Fischer et al., 2015; Zhao et al., 2016; Bornes et al., 2019). In a breast cancer lineage tracing model recently published by Lüönd et al. (2021) the authors demonstrated that partial EMT cells, but not full EMT cells, are required for lung metastasis, while both contribute to the development of chemo-resistance. Interestingly, the hybrid epithelial/mesenchymal phenotype was also found to exhibit an immune-suppressive capacity in breast carcinoma models, as demonstrated by Dongre et al. (2021); Sahoo et al. (2021).

BOX 1 **Hallmarks of EMT**

EMT can be induced by various extracellular stimuli such as cytokines belonging to transformation growth factor β (TGF-β) family, hypoxic conditions or matrix stiffness. These activate signaling cascades that regulate structural and functional changes in epithelial cells (Nieto, 2013; Lamouille et al., 2014). Epithelial cells are constituted of sheets of cells that are tightly packed *via* specialized cell-cell junctions. One of which are the cell-cell adhesion junctions that require epithelial cadherin (E-cadherin). Upon EMT cells undergo a “Cadherin-switch,” whereby E-cadherin is downregulated and replaced by neural-cadherin (N-cadherin) (Christofori, 2006). This switch is directly linked to the loss of cell-cell adhesions, activation of EMT regulatory pathways (e.g., Wnt signaling) and rearrangement of the cytoskeleton. The cortical actin is typical to epithelial cells and is reorganized to form stress fibers. The epithelial apical-basal polarity is essential to their function, and is lost during EMT, resulting in front-rear polarity and fibroblast-like morphology (Yilmaz and Christofori, 2009). These major morphological changes are the result but also the cause of transcription factor activation and EMT-associated signaling regulation. Transcription factors regulating EMT, such as ZEB, Snail and Twist, are tightly controlled at the post-transcriptional level by various micro-RNAs (miRNAs) (Lamouille et al., 2014). Members of the miR-200 family are associated with epithelial cell morphology and their expression is decreased upon the induction of an EMT. ZEB1 and ZEB2 directly bind to miR-200 promoters and repress their expression, in turn miR-200 repress ZEB1/2. A number of such double negative feedback loops between miRNAs and key EMT TFs have been described. These negative feedback loops function as molecular switches and are important mechanisms underpinning the fine-tuning and reversibility of EMT and, thus, epithelial/mesenchymal cell plasticity (Brabletz, 2012; Diepenbruck and Christofori, 2016).

### 2.2 Cancer stem cells

Although broadly studied, cancer stem cells (CSCs) are ill defined. This has to do with the elusive definition of stemness and possibly the transient dynamic nature of these cells (Clevers, 2011; Pattabiraman and Weinberg, 2014; Koren and Bentires-Alj, 2015; Laplane, 2017). A key question in the CSC field refers to the cell-of-origin of a tumor (Beck and Blanpain, 2013); is cancer a disease originating from a transformed stem cell (hierarchical model) (Pardal et al., 2003) or do CSCs originate from non-stem cancer cells (Koren and Bentires-Alj, 2015)? A further plausible variation is that oncogene activation can directly induce stemness in non-stem cells (Koren et al., 2015). Cancer types, like teratomas or APL, seem to fit the hierarchical model (Clevers, 2011). However, tumors originating from epithelium (carcinomas) are able to undergo dedifferentiation processes like EMT, resulting in enhanced tumorigenesis potential, demonstrating that CSCs could originate from non-stem cells (Lamouille et al., 2014). A book by Lucie Laplane, entitled “cancer stem cells: philosophy and therapy” represents a deconvolution of the concept of CSC (Laplane, 2017); Laplane suggests a new definition to the concept of stemness divided into four versions: Categorical: stemness is an intrinsic property of a stem cell, independent of its environment; Dispositional: stemness is essential to stem cells but the expression of stemness depends on extrinsic factors; Relational: stemness is an extrinsic property induced in a cell that would otherwise be a non-stem cell; and Systemic: stemness is an extrinsic property of a system such as tissue. Taken together the complexity and dynamics of CSCs, with cellular plasticity contemporaneous to differentiation state, it is possible that targeting CSCs alone will not inhibit cancer plasticity.

### 2.3 Metastatic dissemination

During the journey of metastatic dissemination, cancer cells face a frequently changing microenvironment. Disseminating cancer cells are required for constant adaptation in order to survive blood circulation, seeding and eventually proliferating at distant organ sites (Brabletz, 2012; Diepenbruck and Christofori, 2016; Massagué and Obenauf, 2016). The recruitment of stromal and immune cells to the tumor cells changing microenvironment induces EMT and MET (Labelle et al., 2011; Gao et al., 2012a). EMT and dormancy-induced growth arrest imposes further challenge on the cancer cells during colonization. Thus, re-proliferation at metastasis site requires re-differentiation *via* MET (Gao et al., 2012b; Ocaña et al., 2012; Tsai and Yang, 2013). Interestingly, single cell analysis of a metastatic signature in triple-negative (ER−/PR−/HER2−), basal-like patient-derived xenograft (PDX) models revealed that early-stage metastatic cells expressed a distinct basal/stem-cell signature with upregulation of pluripotency genes as well as EMT markers (Lawson et al., 2015). Thus, raising the possibility that dedifferentiation induced by EMT/MET is a mechanism exploited also by aggressive mesenchymal-like cancer subtypes.

### 2.4 Treatment evasion

Cancer evasion from therapy represents a major hurdle on treatment success, frequently resulting in cancer progression and relapse. One of the main strategies applied by both resistant and tolerant cancer cells is the exploitation of the EMT process (Boumahdi and de Sauvage, 2020). EMT contributes to drug resistance in two ways; *a priori*, when cancer cells undergoing EMT evade therapeutic strategies, and a posteriori when cancer cells adapt to therapy by undergoing EMT. For example, TGF-β-responding cancer cells can undergo partial or full EMT leading to the development of drug-resistance (Oshimori et al., 2015; Katsuno et al., 2019). Furthermore, in response to therapy, cancer cells can undergo EMT like changes resulting in enhanced DNA-damage repair, resistance to apoptosis, altered drug metabolism and secrete cytokines leading to immunosuppressive microenvironment (Holohan et al., 2013; Aiello and Kang, 2019). As mentioned above, the contribution of both partial and full EMT to the development of chemoresistance in breast cancer has been recently demonstrated (Lüönd et al., 2021). The importance of cell plasticity as a mechanism of treatment tolerance was highlighted in clinical cases of non-small cell lung cancer (NSCLC) treated with epidermal growth factor receptor (EGFR) TKIs, transformed into small-cell lung cancer (SCLC). Intriguingly, a potential reversibility of the process was demonstrated when some of these patients regained sensitivity to the treatment following a drug holiday (Sequist et al., 2011). This reversibility was attributed to non-mutational mechanisms of treatment escape, also referred to as drug tolerance and persistence. It has also been shown that drug tolerant persister cells constitute a quiescent reservoir that can eventually give rise to a heterogeneous resistant population (Ramirez et al., 2016; Boumahdi and de Sauvage, 2020). Considering the reversibility, dynamicity and the non-genetic nature of both EMT and drug-tolerance processes, the role of EMT as one of the strategies taken by tolerant cancer cells becomes apparent (Shen et al., 2020). Aldonza and colleagues observed the upregulation of EMT markers in persistent human epithelial lung cancer cells to both Paclitaxel and EGFR tyrosine kinase inhibition treatment (Aldonza et al., 2020). In breast cancer, it has been shown that MEK and PI3K/mTOR inhibitor-driven basal-like persistent cells in humans develops through EMT-related cell state transitions (Risom et al., 2018). Additionally, an acquisition of mesenchymal identity in HER2-amplified breast cancer cells upon treatment with Lapatinib was demonstrated (Hangauer et al., 2017). Following these discoveries, efforts were made to detect and target the vulnerabilities of drug-tolerant cells, in various cancers and treatments (Hangauer et al., 2017; Chauvistré et al., 2022). A recently published work identified a unique sub-population of cycling persister cells that can potentially be targeted therapeutically (Oren et al., 2021). Intriguingly, the EMT signature was identified in both cycling and non-cycling persister populations.

### 2.5 Targeting cancer cell plasticity

Plasticity provides cancer cells with increased heterogeneity, treatment escape and metastatic formation, often resulting in treatment failure and cancer relapse (Koren and Bentires-Alj, 2015; Van Keymeulen et al., 2015; Massagué and Obenauf, 2016). Therapeutic approaches to overcome cancer cell plasticity can be broadly divided to three categories; preventing cell plasticity (Gupta et al., 2009; Al-Lazikani et al., 2012; Pecot et al., 2013; Proffitt et al., 2013; Cortez et al., 2014; Smith et al., 2014; Wilson et al., 2014; Meidhof et al., 2015; Zhang et al., 2019; Dudás et al., 2020; Jonckheere et al., 2021), eliminating cells with enhanced plasticity (Gupta et al., 2009; Wilson et al., 2014; Hangauer et al., 2017) and reversing plasticity *via* differentiation of cancer cells into well-differentiated entities (Pattabiraman et al., 2016; Italiano et al., 2018). Detailed discussion of the first two approaches is beyond the scope of this review and is thoroughly discussed by Boumahdi and de Sauvage (2020). Here, we will focus on the concept and strategies of inducing cancer cell differentiation.

## 3 Differentiation therapy

### 3.1 Concept

The notion that cancer stem cells can be induced to undergo differentiation has been suggested by G.B. Pierce in his study of teratomas in 1959 (PIERCE and DIXON, 1959). Pierce established the concept of CSCs and differentiation potential, establishing a crucial milestone in the field of cancer stem cell biology (Arechaga, 2003). His results demonstrate as he describes “cancer cells as a caricature of the normal process of tissue renewal” (Pierce et al., 1977; Arechaga, 2003). This notion implies that all tumors originate from tissue stem cells and that tumors differ only in the potential for differentiation of their stem cells: embryonal carcinomas form the three germ layers, breast cancer stem cells form only glandular epithelium, and stem cells of squamous cell carcinoma of the skin differentiate into well-differentiated squamous cells (PIERCE and DIXON, 1959; Pierce et al., 1977; Coombs et al., 2015). Indeed, in this latter report, Wallace and Pierce demonstrate that the progeny of malignant squamous cells can differentiate into non-proliferating squamous cells incapable of forming a tumor (Pierce and Wallace, 1971).

The application of differentiation therapy on Acute promyelocytic leukemia (APL) has been a tremendous success. APL is a distinct highly malignant subtype of acute myeloid leukemia. It is characterized by a chromosomal translocation, which results in the fusion between the promyelocytic leukemia (PML) gene and the retinoic acid receptor (RAR) gene. Early treatment with chemotherapy was the front-line treatment of APL with limited remission success and low long-term survival rate (Wang and Chen, 2008). A new era in the treatment of this disease began with a differentiation therapy approach initially developed in China. As the authors describe, this new direction in cancer treatment has its origins in disease control models employed in China that had been influenced by the Chinese ancient philosophy on the management of society. These are best illustrated by Confucius’ famous saying: “If you use laws to direct the people, and punishments to control them, they will merely try to evade the laws, and will have no sense of shame. But if by virtue you guide them, and by the rites you control them, there will be a sense of shame and of right.” The translation of this philosophy into cancer therapy in their research was described as “educating” cancer cells rather than killing them (Wang and Chen, 2008). This philosophy led to the introduction of all-trans retinoic acid (ATRA) in APL patients to induce terminal differentiation of the leukemic promyelocytes into mature granulocyte. Further development in this therapeutic strategy by applying arsenic trioxide (ATO) improved the clinical outcome of refractory or relapsed as well as newly diagnosed APL patients. The combination of ATRA and ATO demonstrated synergism in inducing differentiation and apoptosis turning this disease from highly fatal to highly curable (Wang and Chen, 2008; Coombs et al., 2015).

Applying this concept to solid tumors, implies that differentiation therapy will force the cell back to the cell of origin, a well-differentiated ancestor. In carcinomas, dedifferentiated cancer cells (e.g., EMT-derived cells) can revert back to an epithelial state by undergoing MET. In the next section we will discuss breakthroughs and challenges in the development of differentiation therapy for solid tumors.

### 3.2 The challenge

Pierce, Wang, and Chen demonstrated that the application of differentiation-based therapy usually requires an established model of cancer progression, that parallels to the normal development course (Cruz and Matushansky, 2012). This parallelism is evident in APL, as it follows the hierarchical model discussed in the CSC section. In solid tumors, the correlation is less clear as the tumorigenesis process usually involves mutation-driven gradual transformation of benign cells to cancer cells. Furthermore, most of solid tumorigenesis involves multiple oncogenic pathways, in contrast to the single main tumor genetic abnormality in APL, making the diversion to normal much more complex (Vogelstein et al., 2013). The challenge of applying differentiation therapies on solid tumors is further expressed in the fact that many of the tumorigenic differentiation pathways, such as Wnt and TGFβ signaling, are also involved in adult tissue hemostasis, making drug safety a significant limitation on treatment efficacy (Kahn, 2014).

The notion that cancer cell plasticity is an inherent feature of many of the solid tumors impedes on the stability of the outcomes and therefore constitute another hurdle on the success of differentiation therapy. Forcing MET is challenging as there is growing evidence that MET may enhance metastatic outgrowth (Brabletz, 2012; Pattabiraman and Weinberg, 2014). Cancer cells forced to re-differentiate can potentially regain cellular plasticity by undergoing another round of EMT. Moreover, in contrast to broadly accessible blood samples in the case of hematologic malignancies, solid tumor biopsies are harder to acquire to enable in-depth differentiation therapy research (de Thé, 2018).

### 3.3 Affecting cell signaling

#### 3.3.1 Retinoic acid signaling

Prior to the success of ATRA, scientists explored retinoic acid (RA) as propagator of differentiation in solid tumors. RARα is an essential RAR, which upon binding to RA, regulates cell proliferation and differentiation (Garattini and Terao, 2007). In 1982, RA-treated osteosarcoma and chondrosarcoma cells, exhibited reversible growth inhibition and reduced colonization (Thein and Lotan, 1982; Ng et al., 1985). More recent studies showed ATRA induced osteogenic differentiation in osteosarcoma, both *in vitro* and *in vivo* (Luo et al., 2010; Dingwall et al., 2011; Hisada et al., 2013). Additional ATRA mechanisms have been discovered. For instance, the link to key signaling pathways, such as TGF-β, NF-κB, mitogen-activated protein kinase (MAPK) and Notch signaling pathways (Dingwall et al., 2011; Yang et al., 2012). Additionally, inhibition of M2 polarization of tumor-associated macrophages (TAMs), that was found to prevent metastasis (Zhou et al., 2017). Rhabdomyosarcoma *in vitro* studies demonstrated a decreased proliferation and increased differentiation upon ARTA treatment (Garvin et al., 1986; Crouch and Helman, 1991; Brodowicz et al., 1999; Barlow et al., 2006). In neuroblastoma, a solid pediatric tumor arising from dedifferentiated neuronal cells, which constitute a window of opportunities for pro-differentiating and anti-proliferative therapy, RA is employed in clinical practice as 13-cis RA, also known as Isotretinoin, as part of the treatment for high-risk neuroblastoma. However, many patients do not respond to the treatment, and further research effort, including of combinational therapy, that will be reviewed next, is required (Matthay et al., 1999; Reynolds et al., 2003; Masetti et al., 2012). A synergistic effect on osteosarcoma was observed with ATRA and methotrexate co-treatment (Sramek et al., 2016) and by the combination of ATRA and peroxisome proliferator-activated receptor-γ (PPARγ) agonists (He et al., 2010), another key player that will be discussed in the coming section. In neuroblastoma, synergistic effect between RA and other drugs, such as cellular processes mediators, epigenetic modifiers, and immune modulators (MAP/PI3K/TGF-β agonists and CYP26/PKC/tyrosine kinase/proteosome inhibitors) has been shown to be advantageous (Rössler et al., 2006; Chevrier et al., 2008; Clark et al., 2013; Duffy et al., 2017; Bayeva et al., 2021). Following the emergence of immunotherapy, a combination with anti-GD2 antibodies and IL-2 was introduced, with promising outcomes improving overall survival (Gilman et al., 2009; Yu et al., 2010; Cheung et al., 2012; Siebert et al., 2016; Mueller et al., 2018; Park and Cheung, 2020). Furthermore, there is strong evidence of the synergy between RA and epigenetic modulators, predominantly Histone Deacetylase inhibitors (HDACi), which were shown to induce neuroblastoma differentiation both *in vitro* and *in vivo* (Coffey et al., 2001; De los Santos et al., 2007; Frumm et al., 2013; Almeida et al., 2017; Westerlund et al., 2017; Kolbinger et al., 2018; Lochmann et al., 2018). We will further discuss the usage of HDAC inhibitors in differentiation induction in the following section.

#### 3.3.2 Peroxisome proliferator-activated receptor-γ signaling

PPARγ is a ligand-activated transcription factor that plays an important role in a variety of physiological processes. PPARγ was initially characterized as the master regulator for adipogenesis but PPARγ signaling has also been implicated in the control of cell proliferation and metabolism. Ligands for PPARγ include naturally occurring fatty acids and a class of anti-diabetic drugs, the thiazolidinediones (TZD). Spiegelman and colleagues demonstrated exciting results in various cancer types by manipulating PPARγ (Tontonoz and Spiegelman, 2008). PPARγ is extensively present in malignancies of adipose tissue, liposarcoma, as being a crucial transcription factor in adipocytes (Tontonoz et al., 1997). This observation led the authors to the hypothesis that treating transformed dedifferentiated liposarcomas with TZD would induce a terminal differentiation into benign adipocytes and inhibit tumor progression. Preclinical and clinical experiments demonstrated upregulation of adipocyte markers, reduced proliferation and typical adipocyte morphology in treated liposarcoma tumors (Tontonoz et al., 1997; Demetri et al., 1999; Debrock et al., 2003). Surprisingly, Sarraf and colleagues also observed high PPARγ levels in colon tumors, a cancer type originating from transformed epithelial cells. Thus, they tested the effect of TZD in colon cancer cells demonstrating here as well reduced cancer cell proliferation and increased differentiation. The effect of TZD on colon cancer cells resulted in re-differentiation into colonic epithelial cells with decreased tumorigenic characteristics (Sarraf et al., 1998). Relatively high levels of PPARγ were also found in metastatic breast cancer cells. Here the combination of PPARγ agonists with a MEK inhibitor resulted in decreased proliferation and upregulation of epithelial markers in *in vitro* experiments (Mueller et al., 1998). Additional pre-clinical study showed that TZD induced cell cycle arrest and apoptosis in bladder cancer cells, leading to inhibition of cell proliferation *in vitro* and suppression of tumor growth *in vivo* (Lv et al., 2019). Since this pioneer work on the effects of PPARγ ligands in cancer, multiple clinical studies in various cancer types were conducted. The majority of these studies were done in advanced stage disease and mostly as monotherapy using TZD. Yet, most of these studies did not show a significant clinical benefit (Hatton and Yee, 2008).

#### 3.3.3 Wnt signaling

Wnt signaling has long been implicated in carcinogenesis, metastatic dissemination, and cancer stemness (Malladi et al., 2016; Nguyen et al., 2009; Yu et al., 2012; Tammela et al., 2017). Wnt is also a known EMT transcription factor as previously stated. With the aim of differentiation of solid tumor cancer cells, vantictumab (OMP-18R5), a monoclonal antibody against Frizzled (FZD) receptors 1, 2, 5, 7, and 8 and inhibits canonical WNT signaling, was developed (Gurney et al., 2012). In a human cancer cell line and patient-derived xenograft models of breast cancer, vantictumab treatment resulted in tumor growth inhibition (Gurney et al., 2012; Fischer et al., 2017). A down regulation of gene expression programs associated with EMT was also observed (Gurney et al., 2012; Fischer et al., 2017). In a following phase Ib clinical trial, vantictumab in combination with paclitaxel in patients with locally advanced or metastatic HER2-negative breast cancer had shown decent tolerance and promising efficacy (Diamond et al., 2020). A phase 1b study of the combination of vantictumab with nab-paclitaxel and gemcitabine in patients with previously untreated metastatic pancreatic cancer was limited by bone-related toxicities, which requires further inquiries (Davis et al., 2020). Additional targeting of Wnt signaling is applied in the case of inhibition of a single tumorigenic driver, a rare therapeutic opportunity in solid tumors, as previously mentioned; Storm and colleagues targeted a Wnt pathway component, RSPO3, in PTPRK-RSPO3-fusion positive colon tumors xenografts, which resulted in the initiation of differentiation, loss of stem cell function and inhibition of tumor growth (Storm et al., 2016).

### 3.4 Metabolic reprogramming

Glioblastoma metabolic research have shown that cyclic adenosine monophosphate (cAMP) can induce an anti-Warburg effect, a metabolic shift from aerobic glycolysis to oxidative phosphorylation, that promotes the differentiation of glioblastoma into benign astrocytes (Xing et al., 2017). Pattabiraman et al. (2016), aimed to induce MET in breast cancer cells, found that cAMP activation induced CDH1 upregulation (the gene encoding for E-cadherin) and hence the acquirement of epithelial characteristics. The study further demonstrated a role for the cAMP-downstream effector protein kinase A (PKA) in inducing MET and maintaining an epithelial state (Pattabiraman et al., 2016). The modulation of cAMP signaling pathway, as a key regulator of metabolism, cell proliferation, and differentiation, is being further investigated. An additional metabolic aspect that has been related to differentiation is cholesterol metabolism. While a complex balance between cancer promotion and suppression has been attributed to cholesterol (Silvente-Poirot and Poirot, 2014), it has been shown that dendrogenin A, a selective inhibitor of cholesterol epoxide hydrolase induced tumor re-differentiation and growth control in animal models (De Medina et al., 2013). Interestingly, accumulating data demonstrate an interdependent relationship between metabolic reprogramming and epigenetic mechanisms in cancer cells, among which, metabolism-reaction intermediates are required for the activity of chromatin-modifying enzymes (Kinnaird et al., 2016).

### 3.5 Affecting epigenetic mechanisms

#### 3.5.1 Chromatin modifications

Histone deacetylation, mediated by histone deacetylases (HDACs) is part of epigenetic control of the transcription process. HDAC induces chromatin modifications that modulate transcriptionally repressive “closed” heterochromatin (Siddiqi et al., 2010). Chromatin modifications are essential for development and differentiation. In fact, the association between histones and differentiation is known for several decades, even prior to chromatin modifications recognition (Ru-chih and Bonner, 1962). These modifications can induce malignant transformation by transcriptional repression of tumor suppressors involved in cell growth regulation and differentiation (Mai et al., 2005; Cress and Seto, 2000). HDACi are chromatin-modifying agents known as inducers of cellular differentiation since 1979 (Ebert and Malinin, 1979). Treatment with HDACi successfully produced differentiation in preclinical models of sarcoma, lung and prostate tumors (Supplementary Table S1) (Marks et al., 2001; Wang et al., 2001; Hrzenjak et al., 2006; Platta et al., 2007; Rephaeli et al., 2005; Belinsky et al., 2003). In some carcinomas such as breast and pancreas malignancies, some HDACi were able to induce MET. Mesenchymal invasive cells from mammary gland carcinomas were induced to differentiate into epithelial cells by treatment with SAHA, a specific HDACi. Treatment with SAHA reduced proliferation and induced differentiation in these cells (Supplementary Table S1) (Munster et al., 2001; Kumagai et al., 2007). Mocetinostat, a different HDACi was shown to induce differentiation and to increase sensitivity to chemotherapy in EMT-derived pancreatic cancer cells (Meidhof et al., 2015). Indeed, numerous clinical trials with HDACi have been performed for the treatment of different cancer types. However, HDACi seemed to have conflicting effects on regulating cell-state transitions, and clinical results did not meet the expectations from this class of drugs (Supplementary Table S1) (Tam and Weinberg, 2013). Furthermore, an important point to consider when applying HDACi to the clinic, is the adverse events potential, due to their pleiotropic cell functions. Another chromatin modification leading to differentiation is the inhibition of EZH2, a histone methyltransferase. Histone methyltransferase regulates gene transcription by controlling the access of transcription factors to DNA. EZH2 is frequently overexpressed in both hematological malignancies and solid tumors. Its abnormal activity facilitates modification in cell fate decisions, proliferation, differentiation, and cell migration, including regulation of CDH1 expression. EZH2 was shown to be required for MET during human induced pluripotent stem cells generation (Supplementary Table S1) (Rao et al., 2015; Barsotti et al., 2015). Pre-clinical data in multiple solid tumors suggest a therapeutic role for EZH2 inhibitors (Wee et al., 2014; Keilhack and Smith, 2015), whereas in clinical trials, including patients with non-Hodgkin’s lymphoma (NHL) and solid tumors, only NHL patients responded clinically to the treatment (Ribrag et al., 2015).

#### 3.5.2 Inhibition of DNA methylation

5-Aza-2′-Deoxycytidine (Decitabine) is a specific inhibitor of DNA methylation. DNA demethylation results in differentiation, growth inhibition, and loss of clonogenicity. Decitabine is an approved treatment for myelodysplastic syndrome (MDS) and for acute myeloid leukemia (AML). It’s potency was also shown in animal models and in preliminary clinical trials of NSCLC patients (Momparler, 2005). Later on, an inhibition of tumor growth and induction of melanocyte differentiation of murine melanoma models *in vivo* was demonstrated (Alcazar et al., 2012). In the past few years, decitabine has been developed as an anti-osteosarcoma. Treatment of osteosarcoma cells with decitabine was found to induce ERα expression, decrease proliferation and metastasis-associated markers, and cause osteoblast differentiation (Supplementary Table S1) (Osuna et al., 2019). Ruh et al. (2021) recently demonstrated that Decitabine can induce the demethylation of imprinted DLK-DIO3 locus resulting in the downregulation of ZEB1 *via* microRNAs (miRNAs) expression in osteosarcoma cells. The reduction of ZEB1 expression levels induced an adipogenic and osteogenic differentiation in the cells, as well as improved the response to doxorubicin. Among other EMT transcription factors, ZEB1 has been targeted *via* miRNA at the post-transcriptional level, with the aim of targeting cell plasticity, as mentioned in the plasticity section.

### 3.6 Targeting epithelial-mesenchymal transition feedback loops

Computational and experimental analysis have examined the dynamics of the regulatory networks involved in EMT and discovered the importance of feedback loops (Brabletz and Brabletz, 2010; Siemens et al., 2011; Craene and Berx, 2013; Lu et al., 2013; Tian et al., 2013; Shi et al., 2014; Jolly et al., 2015a; Jolly et al., 2015b; Kundu et al., 2016; Jia et al., 2017; Mooney et al., 2017; Celià-Terrassa et al., 2018; Jolly et al., 2018; Hari et al., 2020; Silveira and Mombach, 2020). Such feedback loops usually consist of both epithelial and mesenchymal players, each regulating the expression of the other. The balance between the two dictates the cell state (Gregory et al., 2011; Jolly et al., 2015a; Diepenbruck et al., 2017). Understanding feedback loops in EMT regulation and the acknowledgment of the hazardous potential in cancer plasticity, allowed the development of a new therapeutic approach also targeting phenotypic plasticity (Brabletz and Brabletz, 2010; Lu et al., 2013; Tian et al., 2013; Diepenbruck et al., 2017; Jia et al., 2017; Mooney et al., 2017; Celià-Terrassa et al., 2018; Jolly et al., 2018; Hari et al., 2020; Silveira and Mombach, 2020). Computational analysis of EMT feedback loops found that some can cause the persistence of cancer cell plasticity after stimulation withdrawal, and that the reduction of positive feedback loops in the EMT plasticity network can restrict it (Hari et al., 2020). This idea was preliminary implemented by Celià-Terrassa et al. (2018), who showed a reduction in metastatic dissemination by breaking the miR-200/ZEB loop. Yet, manipulating the inherent switch in feedback loops can be counterproductive in the context of carcinomas; restricting EMT can enhance colonization *via* MET and MET inhibition may propagate dissemination and stabilization of hybrid Epithelial/Mesenchymal state can potentially encourage cancer cell dissemination and treatment escape. The cell plasticity maintained in cancer cells *via* EMT and MET is inherent to various solid cancers, possibly the main hindrance of re-differentiation therapies. This notion gave rise to the need to exit the plasticity loop, resulting in the development of a trans-differentiation approach.

### 3.7 Trans-differentiation therapy—making the problem an opportunity

A possible explanation for the limited clinical gain from reversion of cancer cells back into their original differentiated cell type is the inherent plasticity of the epithelial cancer cells. To overcome the plasticity challenge in cancer treatment, we have recently demonstrated that enhanced cancer cell plasticity can be therapeutically exploited by inducing trans-differentiation into different cell types. EMT-derived breast cancer cells were induced to undergo terminal trans-differentiation into mature non-proliferating adipocytes (Ishay-Ronen et al., 2019). Trans-differentiation is possible in cancer cells since the process of EMT seems to induce multi-potency in cancer cells, enabling cell fate shift. Our results suggest that the differentiation potential is impeded by the activation of MEK-ERK signaling. Thus, the combination of MEK inhibitors to enable differentiation with PPARγ agonist, to induce adipogenesis can result in cancer trans-differentiation into *bona fide* adipocytes (Tontonoz et al., 1997; Demetri et al., 1999; Debrock et al., 2003; Ishay-Ronen and Christofori, 2019; Ishay-Ronen et al., 2019). The breast cancer-derived adipocytes exhibit inherent terminal differentiation and growth arrest, thus lacking cellular plasticity, which practically resulted in the prevention of invasion and metastasis in various *in vivo* models (Ishay-Ronen et al., 2019). These results indicate the potential of utilizing the increased cell plasticity inherent to invasive cancer cells for trans-differentiation therapy.

## 4 Discussion

**FIGURE 3:**
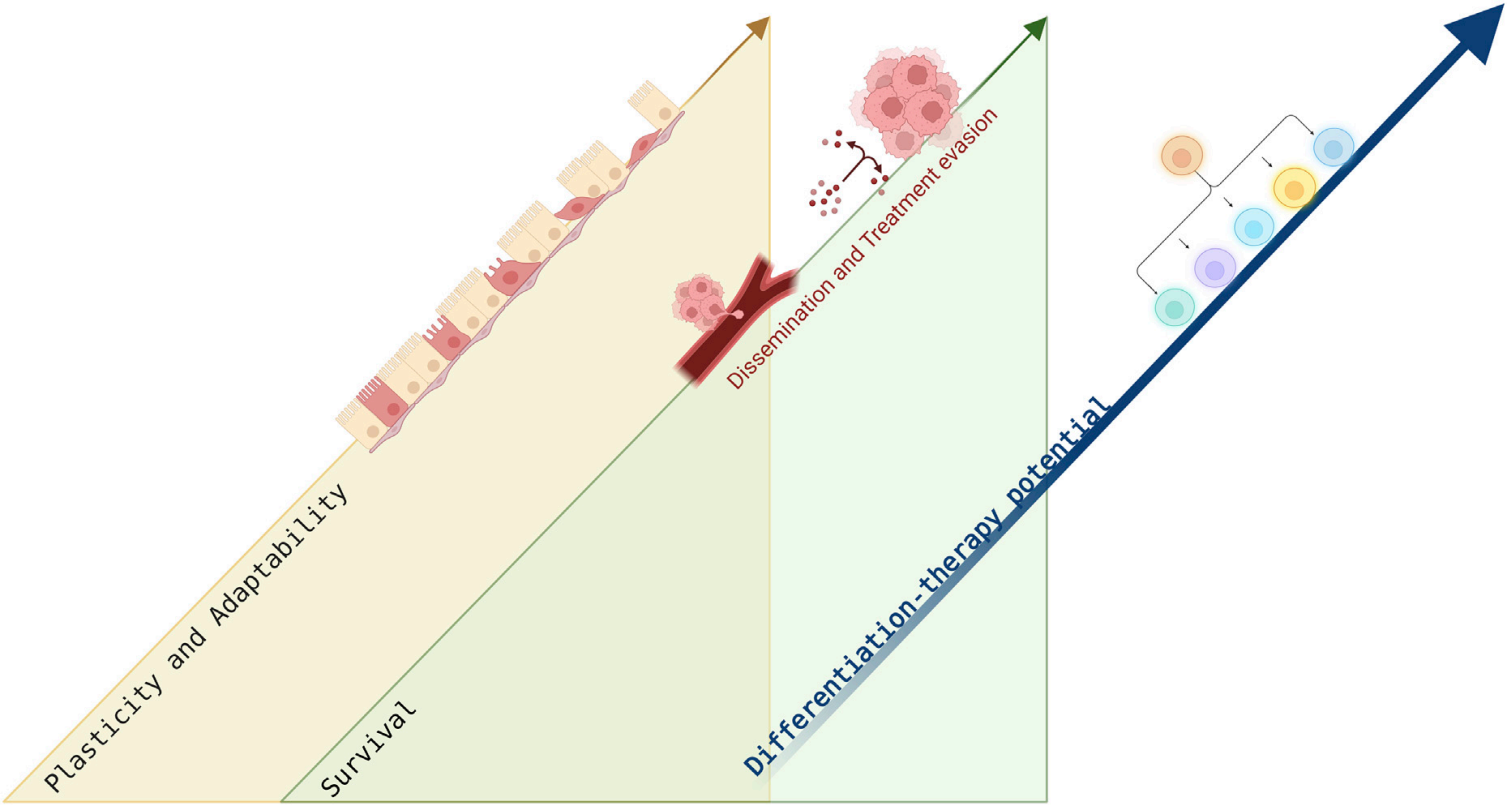
Differentiation treatment potential facilitated by cancer plasticity. This figure schematically illustrates the concurrency of cell plasticity and cancer survival during malignant progression with the differentiation potential. When cancer cells are responding to stress induced by the changing microenvironment during dissemination or in response to treatment application, cell plasticity and adaptability are enhanced, correlating with cell survival. The increased cell plasticity is linked to the multi-differentiation potential of the cells, proposing a therapeutic window. Figure created with BioRender.com.

Plasticity is a quality of the cell that crafts its capacity to adapt to the changing environment. Cellular plasticity can be enhanced during cancer progression, by hijacking EMT and MET programs to promote tumor growth, survival and metastatic dissemination which eventually aggravate patient outcomes (Nieto, 2013). Cancer differentiation state is essential for the histopathological classification of solid malignancies and is strongly associated with tumor behavior and aggressiveness. The great impact of cancer plasticity, and the effect of differentiation state of solid tumors, motivated the quest for pharmacological discoveries to overcome cancer plasticity, raising the possibility to force re-differentiation and trans-differentiation in solid tumors. In our view, plasticity, EMT and MET are processes contemporaneous to the cell differentiation state, which means that the window of plasticity should be exploited in the quest for a proper solution (Figure 3). A trans-differentiation therapeutic approach resulted in irreversible cell state with impeded cell plasticity. The review highlights the therapeutic potential in differentiation approaches to overcome the cellular plasticity inherent to cancer cells. In fact, accumulating data suggests that cell plasticity can be utilized therapeutically with differentiation approaches. Differentiation treatment strategies are unique in their potential to target slow-cycling, dormant, dedifferentiated cancer cells with stem cell—like characteristics. Yet, complex signaling activation and differentiation inhibition induced by cancer progression require further research to uncover mechanisms regulating cancer differentiation. Furthermore, a positive clinical outcome in the context of differentiation treatment is rather obscure since treatment response might not correlate with tumor eradication. It is plausible that differentiation treatment, targeting resistant disseminating cancer cells, can become clinically beneficial only in combination with conventional treatment modalities. Alternatively, in the context of metastatic disease, differentiation treatment can result in minimal residual disease (MRD), impeding cancer progression. Differentiation therapy in solid tumors was initially suggested more than half a century ago. Yet, our understanding today of cellular mechanisms driving cancer progression can be translated into optimization and further development of this fascinating research field.
