# Supplementary material for: Engaging plasticity: Differentiation therapy in solid tumors
Source: Front Pharmacol. 2022 Aug 11;13:944773. doi: 10.3389/fphar.2022.944773 (PMC9410762; doi:10.3389/fphar.2022.944773)
Supplement: Supplementary file 1 [file Table1.docx]

**Supplementary Table 1- Epigenetic affecting drugs**

|  | Agent | Specific Mechanism | Ref. |
| --- | --- | --- | --- |
| Chromatin modifying | Valproate  (Valproic acid) | Class II HDAC inhibition | (139) |
|  | Trichostatin A | Class I and II HDAC inhibition | (140) |
|  | AN‐7  (Butyroyloxymethyl‐diethyl phosphate) | Non-specific HDAC inhibition | (141) |
|  | Vorinostat/SAHA  (Suberoylanilide hydroxamic acid) | Class I, II and IV HDAC inhibition | (143, 144) |
|  | Mocetinostat | Class I HDAC inhibition | (57) |
|  | EZH2 inhibitor | Histone methyltransferase inhibition | (146-150) |
| DNA methylation inhibition | Decitabine  (5-Aza-2′-Deoxycytidine) | DNMT1 depletion | (151-153) |

HDAC - Histone deacetylases, DNMT1- DNA methyl transferase 1
